# Random survival forests identify pathways with polymorphisms predictive of survival in KRAS mutant and KRAS wild-type metastatic colorectal cancer patients

**DOI:** 10.1038/s41598-021-91330-z

**Published:** 2021-06-09

**Authors:** Madiha Naseem, Shu Cao, Dongyun Yang, Joshua Millstein, Alberto Puccini, Fotios Loupakis, Sebastian Stintzing, Chiara Cremolini, Ryuma Tokunaga, Francesca Battaglin, Shivani Soni, Martin D. Berger, Afsaneh Barzi, Wu Zhang, Alfredo Falcone, Volker Heinemann, Heinz-Josef Lenz

**Affiliations:** 1 Division of Medical Oncology, Sharon Carpenter Laboratory, Norris Comprehensive Cancer Center, Keck School of Medicine, University of Southern California, 1441 Eastlake Avenue, Los Angeles, CA 90033 USA; 2 Department of Preventive Medicine, Norris Comprehensive Cancer Center, Keck School of Medicine, University of Southern California, Los Angeles, CA USA; 3 Oncologia Medica 1, Istituto Oncologico Veneto, Istituto Di Ricovero E Cura a Carattere Scientifico, Via Gattamelata, Padua, Italy; 4 Medical Department, Division of Hematology, Oncology and Hematology, Tumor Immunology (CCM), Charité—Universitätsmedizin, Berlin, Germany; 5 Oncologia Medica, Azienda Ospedaliero-Universitaria Pisana, Istituto Toscano Tumori, Via Roma, Pisa, Italy; 6 Department of Medicine and Comprehensive Cancer Center, Ludwig-Maximilians-University Munich, Munich, Germany

**Keywords:** Cancer genetics, Cancer genomics, Cancer models, Gastrointestinal cancer, Colorectal cancer

## Abstract

KRAS status serves as a predictive biomarker of response to treatment in metastatic colorectal cancer (mCRC). We hypothesize that complex interactions between multiple pathways contribute to prognostic differences between KRAS wild-type and KRAS mutant patients with mCRC, and aim to identify polymorphisms predictive of clinical outcomes in this subpopulation. Most pathway association studies are limited in assessing gene–gene interactions and are restricted to an individual pathway. In this study, we use a random survival forests (RSF) method for identifying predictive markers of overall survival (OS) and progression-free survival (PFS) in mCRC patients treated with FOLFIRI/bevacizumab. A total of 486 mCRC patients treated with FOLFIRI/bevacizumab from two randomized phase III trials, TRIBE and FIRE-3, were included in the current study. Two RSF approaches were used, namely variable importance and minimal depth. We discovered that Wnt/β-catenin and tumor associated macrophage pathway SNPs are strong predictors of OS and PFS in mCRC patients treated with FOLFIRI/bevacizumab independent of KRAS status, whereas a SNP in the sex-differentiation pathway gene, DMRT1, is strongly predictive of OS and PFS in KRAS mutant mCRC patients. Our results highlight RSF as a useful method for identifying predictive SNPs in multiple pathways.

## Introduction

Approximately 30–40% of colorectal cancers (CRC) harbor activating mutations in Kirsten Ras (KRAS) proto-oncogene, which encodes for a GTPase transductor protein downstream of epidermal growth factor receptor (EGFR) as part of the RAS/RAF/MAPK pathway^1^. Activating KRAS mutations are an established predictive biomarker for resistance to anti-EGFR therapies in mCRC. Hence, anti-EGFR therapy is currently used alongside chemotherapy to treat mCRC with wild-type KRAS, whereas the standard of treatment for KRAS mutant patients is chemotherapy with anti-vascular endothelial growth factor (VEGF) monoclonal antibody, bevacizumab^2^.

Although KRAS status has predictive utility, its prognostic role in CRC remains controversial. Colorectal carcinogenesis and treatment responses are a result of complex interactions between multiple genes and pathways. The KRAS protein has a non-linear interaction with different upstream mediators, including receptor tyrosine kinases and growth factors^3^, which adds to the complexity of understanding its role in cancer development and developing effective treatments. It is possible that focusing on KRAS mutations alone, without considering intersecting pathways, is creating barriers in understanding its prognostic value.

Our previous work used genome-wide association studies and Cox-proportional hazard (CPH) models to identify significant differences in predicting survival outcomes in mCRC patients from individual clinical cohorts based on genetic polymorphisms in pathways regulating angiogenesis. However, CPH has several restrictions, for it requires restrictive proportional hazard assumptions, and cannot identify unknown non-linear interactions between genetic pathways or incorporate high-dimensional information found in genomic studies^4^. In this study, we apply a machine learning method, random survival forests (RSF), for identifying predictive polymorphisms from multiple pathways.

Random survival forests (RSF) is a non-parametric ensemble tree learning method that has become increasingly popular for genetic and gene expression data analyses^5^. It has been successfully applied to cancer staging and integrative genomic modelling. In contrast to the CPH model, RSF is an automated approach to identify non-linear multivariate effects, even among highly correlated subsets of covariates, which is particularly useful in high-dimensional feature selection problems. An RSF ensemble comprises randomly grown recursively partitioned binary trees. Each tree is grown from an independent bootstrap sample, and during the tree growing process, each node is split using a randomly selected subset of variables. These properties make it an attractive tool for the analysis of complex survival data^4^.

Hence, in this study, we illustrate the utility of random survival forests (RSF) in integrating complex interactions and uncovering polymorphisms in multiple pathways predictive of survival in mCRC patients based on their KRAS status.

## Materials and methods

### Study population

A total of 486 patients with mCRC enrolled in two multi-institutional open-label randomized phase III trials: TRIBE (NCT00719797)^6^ and FIRE-3 (NCT00433927)^7^ were included in the current study. The TRIBE trial compared efficacy of FOLFOXIRI/bevacizumab with FOLFIRI/bevacizumab in both KRAS mutant and wild-type mCRC patients. The FIRE-3 trial compared efficacy of FOLFIRI/cetuximab to FOLFIRI/bevacizumab as first-line treatment in KRAS wild-type mCRC patients. We combined patients from the two cohorts treated with the same regimen: first-line FOLFIRI/bevacizumab and excluded patients in the other arms.

Eligibility criteria of our study included patients with histologically proven colorectal adenocarcinoma, measurable metastatic disease according to Response Evaluation Criteria in Solid Tumors (RECIST) v1.1, and no prior systemic chemotherapy for metastatic disease. Selected patients with samples available for analyzing genomic DNA were eligible for this study: 189 patients with sufficient samples from TRIBE (75% of 253 enrolled patients) and 297 patients with sufficient samples from FIRE-3 (87% of 343 enrolled patients).

All patients signed an informed consent form before enrollment in the randomized trials which included information regarding the use of their blood or tumor tissue to explore relevant molecular biomarkers. The current study complied with the REporting recommendations for tumor MARKer prognostic studies (REMARK). The specimen analysis was approved by the University of Southern California (USC) Institutional Review Board of Medical Sciences and carried out at the USC/Norris Comprehensive Cancer Center in adherence with the Declaration of Helsinki and Good Clinical Practice Guidelines.

### Selected polymorphisms and genotyping

Twenty-seven candidate single nucleotide polymorphisms (SNPs) within genes involved in Wnt (AXIN2, TCF7L2, SOX9, CBP, CTNNB1), angiogenesis (EGFL7, VEGFR1, VEGFR2 RGS5, PDGFRβ, CSPG4, RALBP1), HIPPO (DSCR1), tumor-associated macrophage (TAM) (HRG, CL2, TBK1), tumor budding (CXCR4, MMP2), autophagy (FIP200), EGFR (EPS15, KSR1, KSR2), and sex-differentiation (FOXL2, DMRT1) pathways were selected according to two major criteria: minor allele frequency (MAF) in Caucasians ≥ 10% (www.ensembl.org); and potential role in changing gene function based on literature review and public databases (https://snpinfo.niehs.nih.gov; https://www.ncbi.nlm.nih.gov). Linkage disequilibrium among selected SNPs was identified through SNAP search service (http://archive.broadinstitute.org/mpg/snap/).

Genomic DNA was extracted from whole blood of patients enrolled in TRIBE using the QIAamp DNAeasy Kit (Qiagen) and from formalin-fixed paraffin-embedded (FFPE) tissues of patients enrolled in FIRE-3. DNA extraction procedures were according to the manufacturer's specifications. DNA sequences were analyzed using the ABI Sequencing Scanner version 1.0 (Applied Biosystems). Investigators performing in SNP analyses were blinded to patients' clinical data. Genotyping was successful in at least 90% of samples in each polymorphism analyzed.

### KRAS mutation analysis

In both trials, mutational analysis of KRAS codons 12, 13, and 61 was conducted using a pyrosequencing approach, and analyzed using PyroMark Q24 1.0.9 software^8^. However, FIRE-3 only included patients who were KRAS wild-type at codons 12 and 13^7^.

### Statistical analysis

The clinical endpoints of this study were progression-free survival (PFS) and overall survival (OS). PFS was defined as period from the first day of randomization start to the first observation of disease progression or death from any cause. OS was calculated from the first day of randomization start to the date of death by any cause. Patients were censored at the date of last follow up if there was no event observed. RSF was used to identify potential predictors for OS and PFS in mCRC patients with wild-type or mutant KRAS.

For each RSF model, a survival forest with 1000 trees was constructed, and each tree was drawn from a random bootstrap sample that excluded on average 37% of the analyzed data, called out-of-bag (OOB) data. Each tree was grown starting from a set of randomly selected candidate variables until final node’s size reached a minimum number of events with unique survival times. At each node, random candidate variables were selected, and the brunch was split using the set of variables that maximized the log-rank statistics, to split the brunch these variables. The importance of a variable is measured by minimal depth (MD), which is the depth when the variable first splits within a tree, relative to the root node^9^. The most predictive variables are identified as those with smallest MD values, which means they split the branches close to the tree trunk. The variable selection threshold is defined as the mean of the minimal depth distribution among all forests, classifying variables with minimal depth lower than this threshold as important in forest prediction.

The cumulative hazard function (CHF) was calculated for each tree and then the ensemble CHF was obtained by averaging CHF. Harrell’s concordance index (C-index) using OOB data were used to evaluate the accuracy for each RSF model. The prediction accuracy for the Cox proportional hazard models including top 5 identified SNPs from RSF models was also evaluated by Harrell’s C-statistics using OOB error rate with 1000 bootstrap samples.

## Results

### Patient characteristics

A total of 486 patients were included in this study, and their baseline characteristics are shown in Table 1. Of these, 345 patients were KRAS wildtype, and 141 were KRAS mutant.

**Table 1:** Baseline comparisons between KRAS wildtype and KRAS mutant patients.

	KRAS wildtype (n = 345)		KRAS mutant (n = 141)		*p* value^†^
	*N*	%	*n*	%	
**Cohort**					< 0.001
TRIBE arm A	96	28	93	66	
FIRE3 Bev arm	249	72	48	34	
**Gender**					0.39
Male	220	64	84	60	
Female	125	36	57	40	
**Age (years)**					
Median (range)	63 (29–76)	62 (33–75)			
≤ 65	189	55	83	59	0.41
> 65	156	45	58	41	
**Performance status**					0.004
ECOG 0	212	61	106	75	
ECOG ≥ 1	133	39	35	25	
**Primary tumor site**					0.75
Right-sided colon	84	25	23	27	
Left-sided colon	251	75	63	72	
Unknown*	10		55		
**Primary tumor resected**					0.099
Yes	280	81	105	74	
No	65	19	36	26	
**Adjuvant chemotherapy**					0.39
Yes	60	17	20	14	
No	285	83	121	86	

^†^*p* value was based on Chi-square test.

*Unknown group was not included in the analysis.

### SNPs and outcomes

The allelic frequencies for all polymorphisms were within the probability limits of Hardy–Weinberg equilibrium.

**Figure 1:**
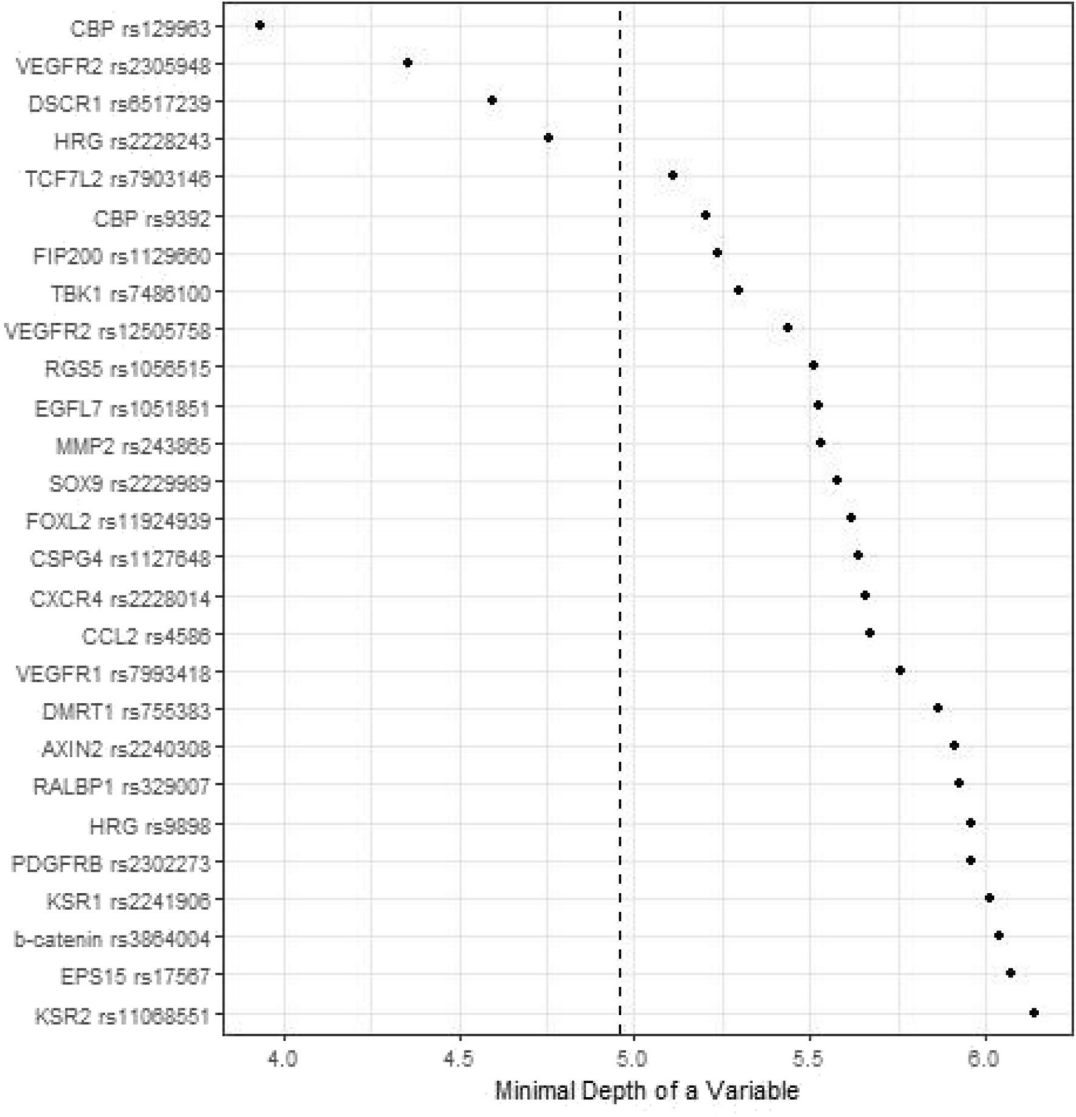
Minimal depth plot of 27 SNPs predicting PFS in KRAS wild-type mCRC patients. The most predictive SNPs in order of importance are CBP rs129963, HRG rs2228243 and TBK1 rs7486100.

**Figure 2:**
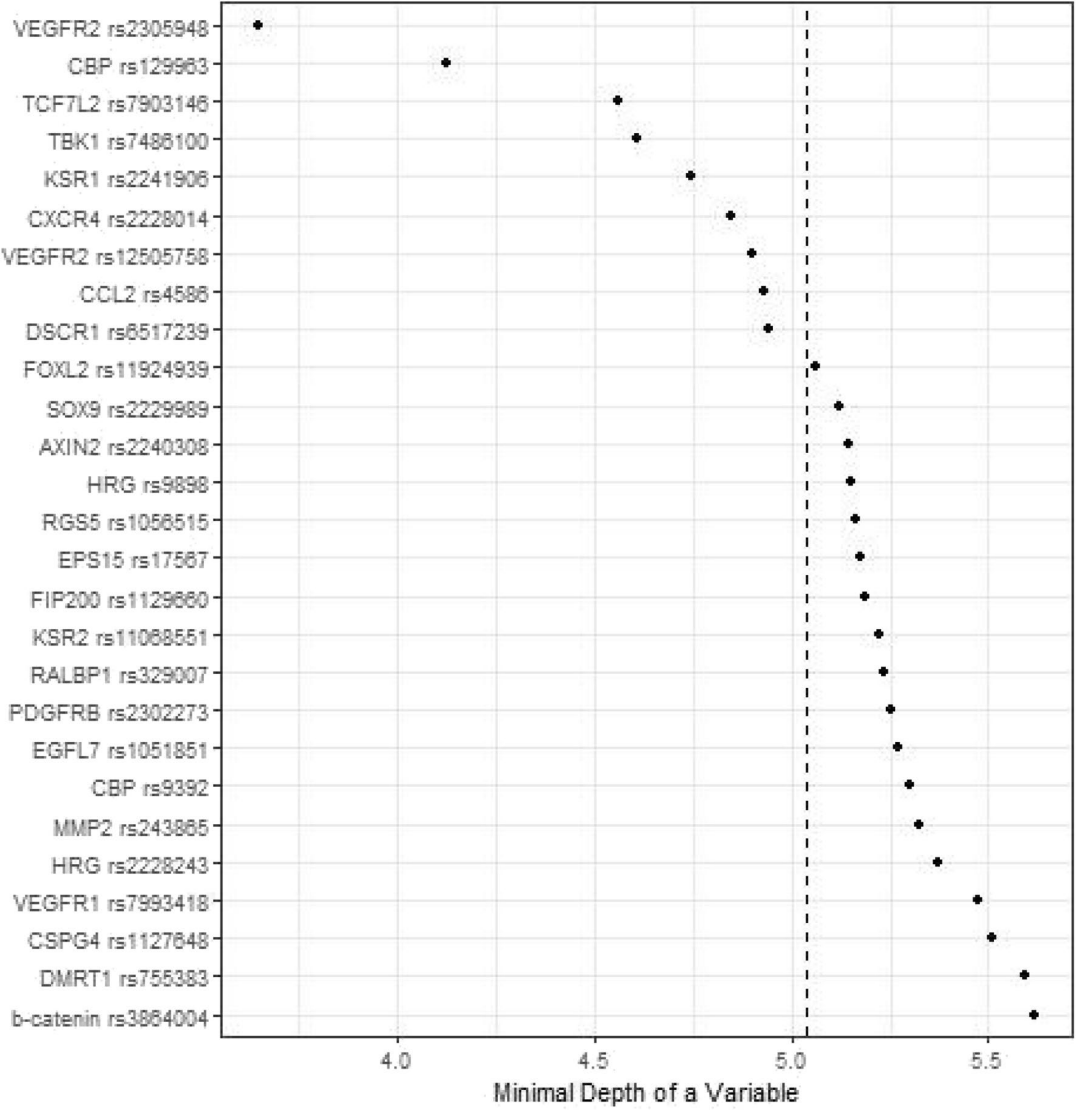
Minimal depth plot of 27 SNPs predicting OS in KRAS wild-type mCRC patients. The most predictive SNPs in order of importance are CBP rs129963, TBK1 rs7486100 and VEGFR2 rs2305948.

**Figure 3:**
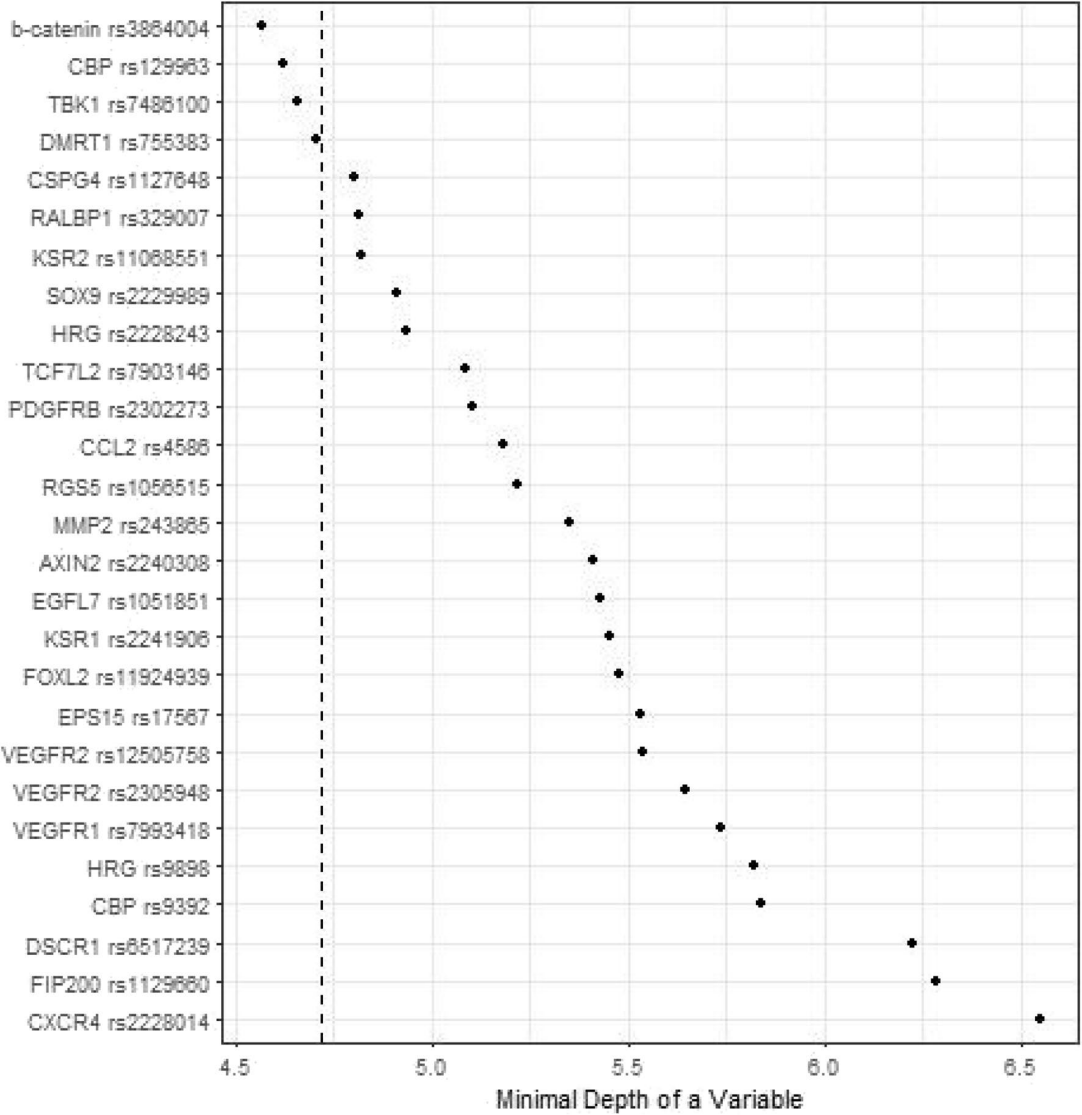
Minimal depth plot of 27 SNPs predicting PFS in KRAS mutant mCRC patients. The most predictive SNPs in order of importance are b-catenin rs3864004, CBP rs129963, TBK1 rs7486100, DMRT1 rs755383.

**Figure 4:**
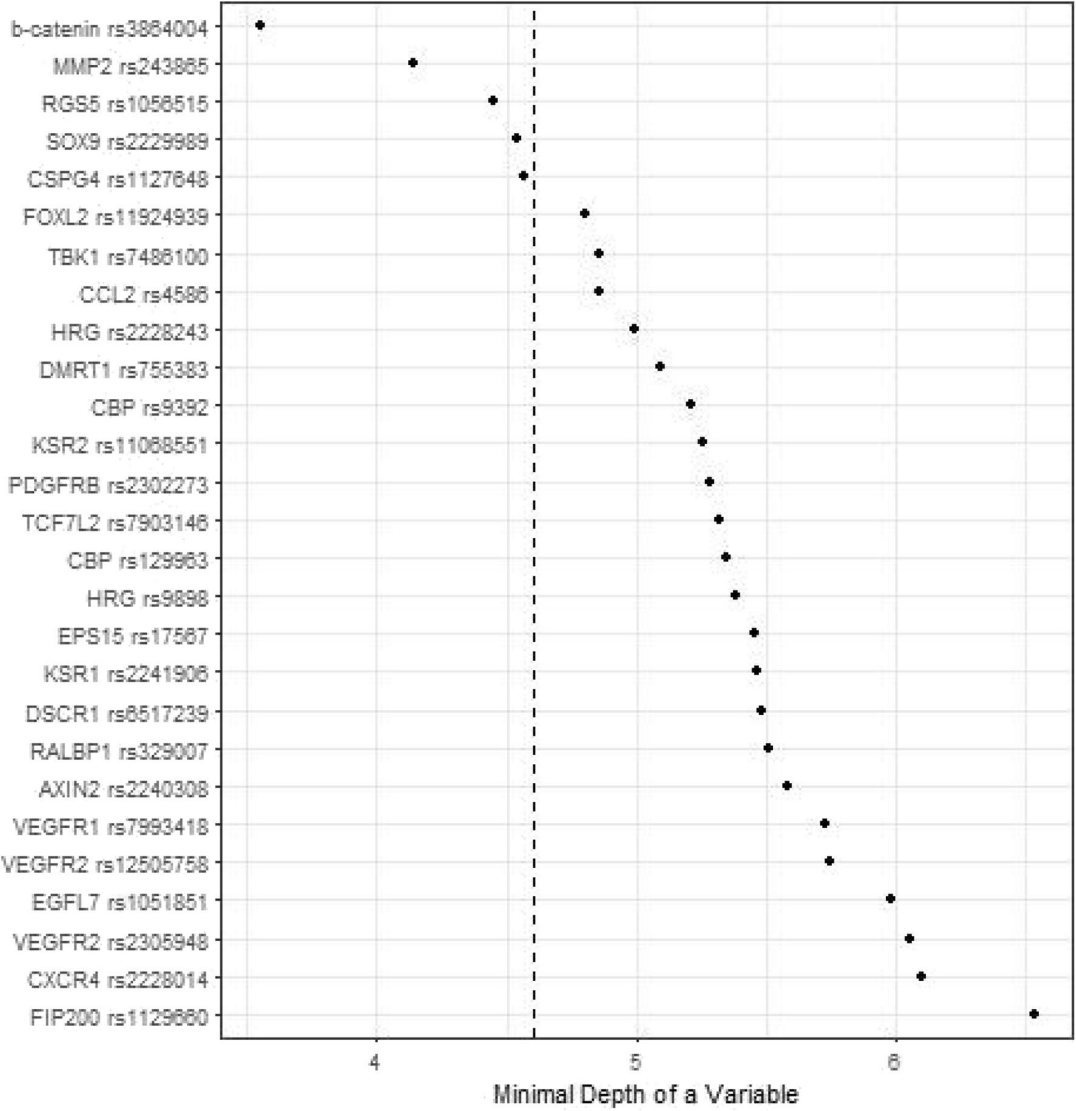
Minimal depth plot of 27 SNPs predicting OS in KRAS mutant mCRC patients. The most predictive SNPs in order of importance are b-catenin rs3864004, MMP rs243865, RGS5 rs1056515, SOX9 rs2229989, and CSPG4 rs1127648.

Figures 1, 2, 3 and 4 depict minimal depth analysis of the 27 SNPs analyzed for PFS and OS in KRAS wildtype and mutant patients. The dashed vertical line in each figure is the threshold of maximum value for variable selection and separates the predictive markers from the remaining non-predictive markers. Low minimal depth indicates important markers. In the KRAS wildtype patients, three SNPs with minimal depth are *CBP* rs129963, *HRG* rs2228243, and *TBK1* rs7486100, which are obviously to be the most predictive markers for PFS. Whereas, *CBP* rs129963, *TBK1* rs7486100, and *VEGFR2* rs2305948 are the most predictive markers for OS. C-index were 0.50 and 0.44 for PFS and OS respectively. In KRAS mutant patients, *β-catenin* rs3864004, *CBP* rs129963, *TBK1* rs7486100, and *DMRT1* rs755383 are the top predictors for PFS; *β-catenin* rs3864004, *MMP2* rs243865, and *RGS5* rs1056515 are most predictive for OS. C-index were 0.55 and 0.45 for PFS and OS, respectively. To compare the RFS model to a Cox proportional hazard models, the top 5 important SNPs selected from each RSF model were used to build a Cox model. In KRAS wildtype patients, the C-index for the Cox models was 0.32 for PFS, and 0.39 for OS; in KRAS mutant patients, the C-indexes were 0.55 and 0.33 for PFS and OS, respectively.

### Compare identified SNPs with previous findings by pathways

All top 5 identified SNPs are summarized in Table 2 according to their pathways. The numbers indicate their ranks from minimal depth results.

**Table 2:** Summary for RSF results by KRAS status.

Pathways	SNPs	KRAS Wildtype		KRAS Mutant	
		PFS	OS	PFS	OS
WNT	AXIN2 rs2240308				
	TCF7L2 rs7903146	5			
	SOX9 rs2229989				4
	CBP rs9392				
	CBP rs129963	1	1	2	
	β-catenin rs3864004			1	1
Angiogenesis	EGFL7 rs1051851				
	VEGFR1 rs7993418				
	VEGFR2 rs12505758				
	VEGFR2 rs2305948		3		
	RGS5 rs1056515	4			3
	PDGFRB rs2302273				
	CSPG4 rs1127648			5	5
	RALBP1 rs329007				
TAM	HRG rs9898				
	HRG rs2228243	2			
	CCL2 rs4586		5		
	TBK1 rs7486100	3	2	3	
Tumor budding	CXCR4 rs2228014				
	MMP2 rs243865				2
EGFR	EPS15 rs17567				
	KSR1 rs2241906		4		
	KSR2 rs11068551				
Y chromosome-related	FOXL2 rs11924939				
	DMRT1 rs755383			4	

#### Wnt PATHWAY SNPs

**Figure 5:**
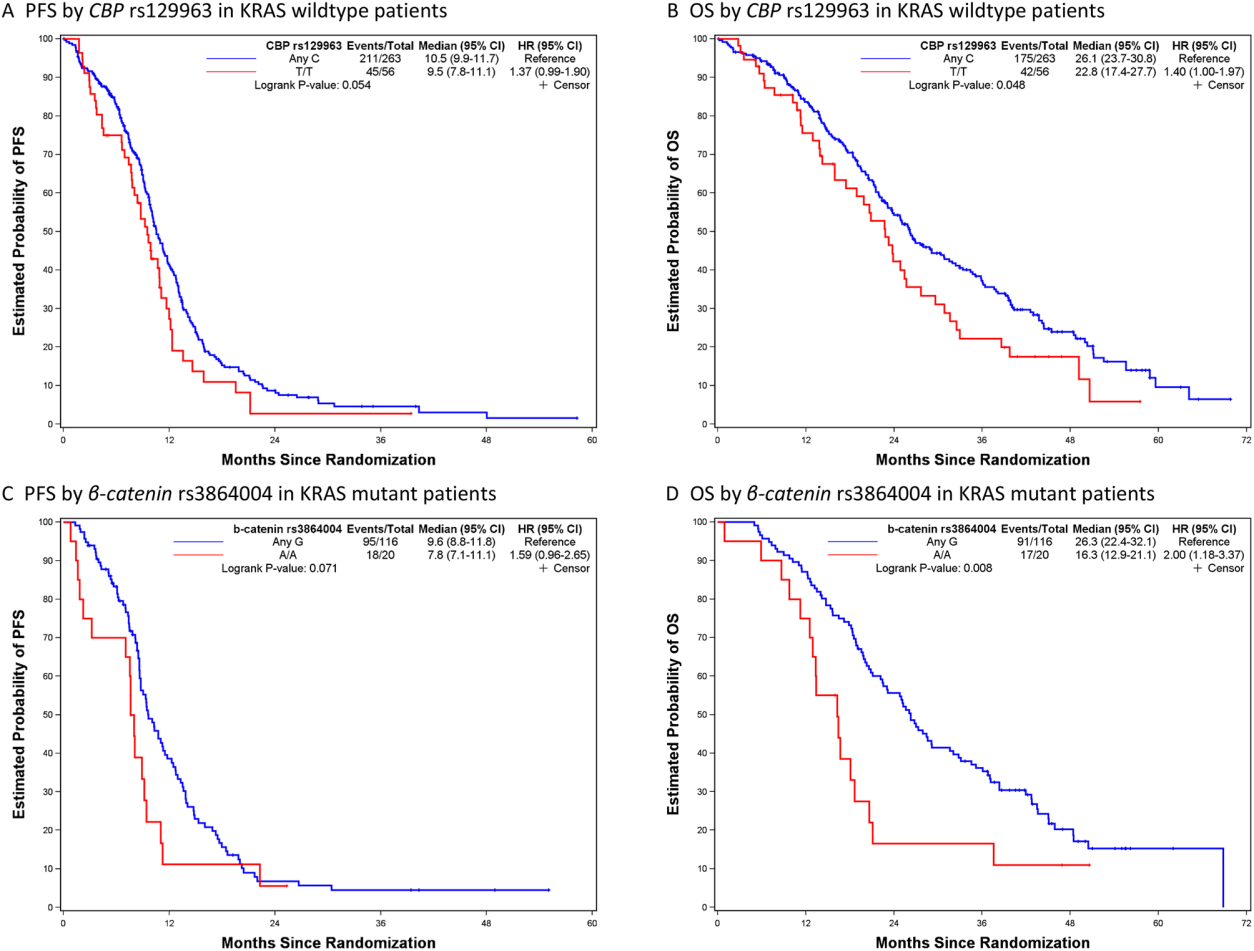
Kaplan–Meier curves and log-rank test for Wnt SNPs predictive of PFS and OS in KRAS wild-type and mutant mCRC patients: (**A**) KRAS wildtype patients with CBP rs129963 T/T variant have shorter PFS (9.5 vs. 10.5 mo; *p* = 0.054) and (**B**) OS (22.8 vs. 26.1 mo; *p* = 0.048) compared to those with Any C allele. (**C**) KRAS mutant patients with β-catenin rs3864004 A/A genotype have shorter PFS (7.8 vs. 9.6 mo; *p* = 0.071) and (**D**) OS (16.3 vs. 26.3 mo; *p* = 0.008).

Previously published work from our lab using CPH models did not identify significant associations between Wnt pathway SNPs and OS or PFS in mCRC patients treated with FOLFIRI/bevacizumab in individual clinical trials^10,11^. Using RSF MD analysis in the current study of combined FOLFIRI/bevacizumab arms from TRIBE and FIRE3 trials, CBP rs129963 and β-catenin rs3864004 are shown to be most predictive markers for OS in KRAS wildtype and mutant patients, respectively. The non-parametric analysis Kaplan–Meier plot and log-rank test for Wnt SNPs are shown in Fig. 5. KRAS wildtype patients with CBP rs129963 T/T variant showed significantly shorter OS compared to those with Any C allele (*p* = 0.048). KRAS mutant patients harboring β-catenin rs3864004 A/A genotype also showed significantly shorter OS (*p* = 0.008).

#### Tumor-associated macrophages (TAM) pathway SNPs

**Figure 6:**
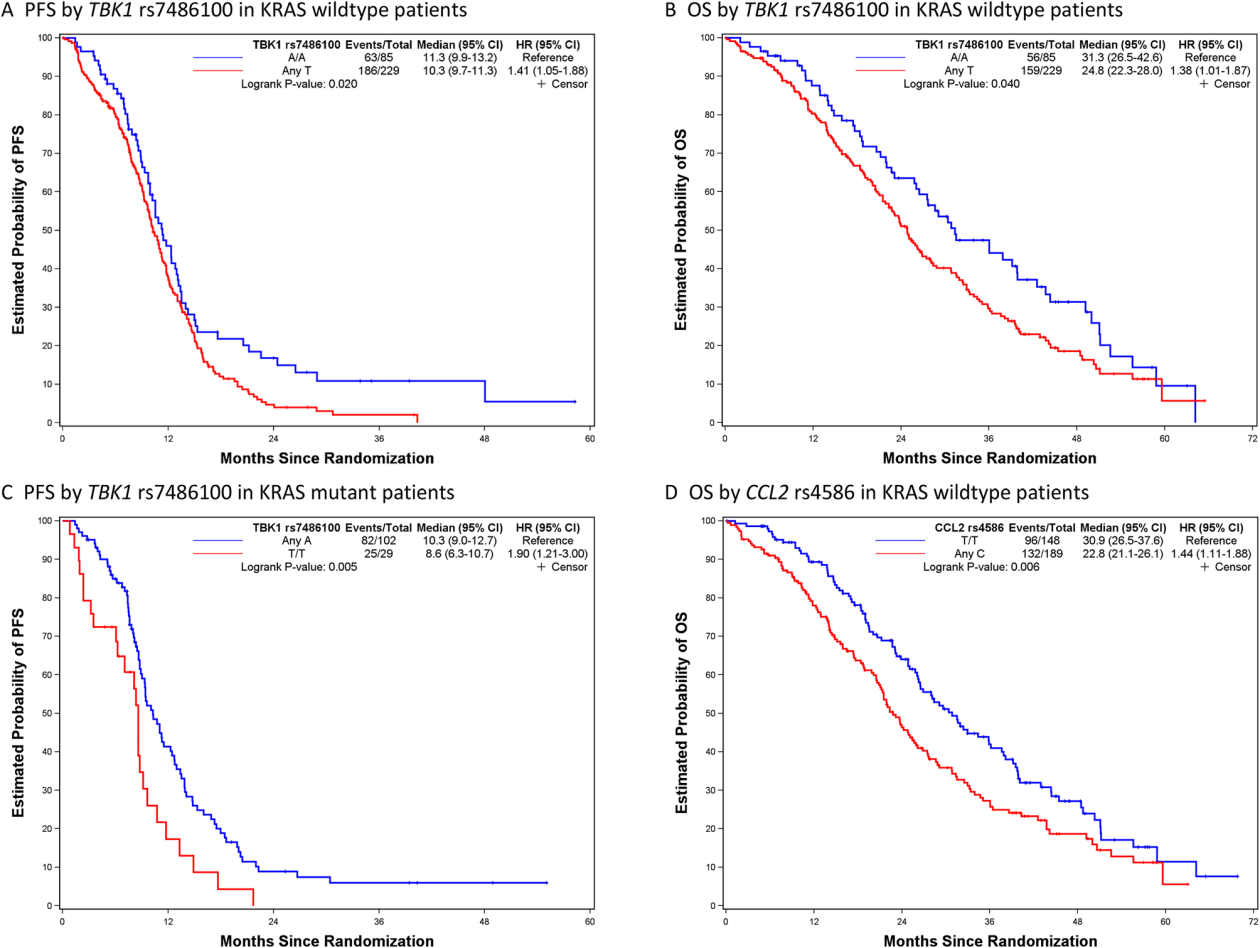
Kaplan–Meier curves and log-rank test for TAM pathway SNPs predictive of PFS and OS in KRAS wild-type and mutant mCRC patients: (**A**) KRAS wildtype patients with TBK1 rs7486100 A/A variant have longer PFS (11.3 vs. 10.3 mo; *p* = 0.020) and (**B**) OS (31.3 vs. 24.8 mo; *p* = 0.040) compared to those with Any T allele. (**C**) KRAS mutant patients with TBK1 rs7486100 A/A genotype also have longer PFS (10.3 vs. 8.6 mo; *p* = 0.005). (**D**) TAM pathway SNP CCL2 rs4586 T/T carriers have longer OS (30.9 vs. 22.8 mo; *p* = 0.006).

RSF analysis identified *TBK1* rs7486100 to be a strong predictor for PFS and OS in KRAS wildtype patients, and for PFS in KRAS mutant patients. In addition, *HRG* rs2228243 and *CCL2* rs4586 are also top predictors for PFS and OS, respectively, in KRAS wildtype patients. Figure 6 showed the Kaplan–Meier plots for TAM SNPs. Wildtype patients with any T allele of *TBK1* rs7486100 showed shorter PFS and OS (*p* = 0.020 and 0.040, respectively); whereas mutant patients with T/T variant had shorter PFS (*p* = 0.005). Although *HRG* rs2228243 was identified as a top marker for OS in KRAS mutant patients, the log-rank test was not significant (*p* = 0.45, plot not shown). Furthermore, the wildtype patients with Any C allele had shorter OS compared to those with T/T variant (*p* = 0.006). From our previous work, *TBK1* rs7486100 was shown to predict OS in KRAS mutant, and *CCL2* rs4586 to predict PFS in KRAS wildtype patients from single arm analysis^12^.

#### Angiogenesis pathway SNPs

**Figure 7:**
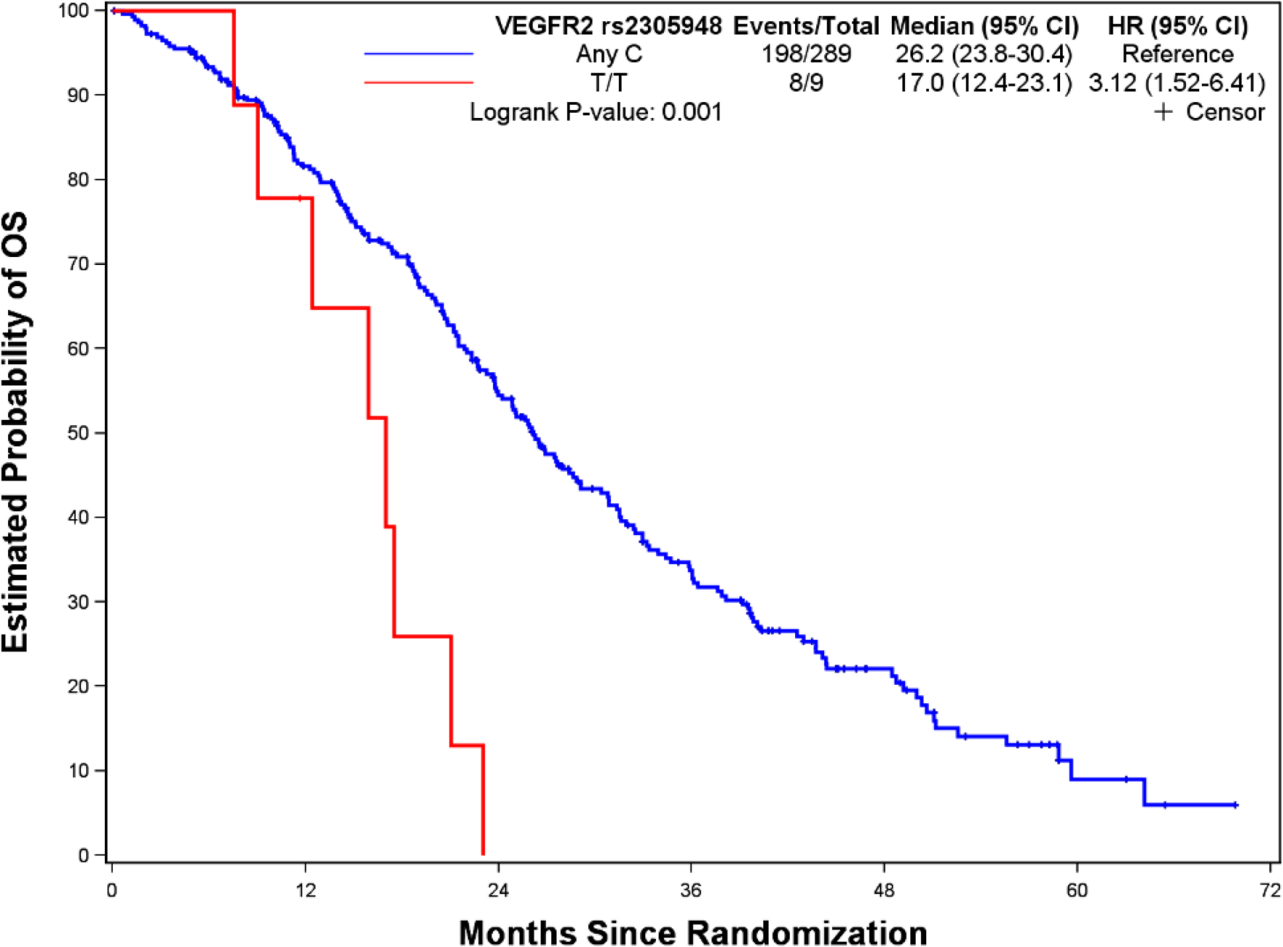
Kaplan–Meier curve and log-rank test for angiogenesis pathway SNP, VEGFR2, predictive of OS in KRAS wild-type mCRC patients. Patients with VEGFR2 rs2305948 Any C carriers have significantly longer OS (26.2 vs. 17.0 mo; *p* = 0.001) compared to T/T carriers.

The results showed *VEGFR2* rs2305948 as a top predictor for OS in wildtype patients. KM plot showed that patients with T/T allele had shorter OS than those with Any C allele (*p* = 0.001) (Fig. 7). Although *RGS5* rs1056515 was also one of top identified SNPs for OS in mutant patients, log-rant test was not significant (*p* = 0.16, plot not shown). Previous study performed in TRIBE and PROVETTA didn’t identify these SNPs, subgroup analysis by KRAS mutation status was not conducted either.

#### Sex-differentiation pathway SNP

**Figure 8:**
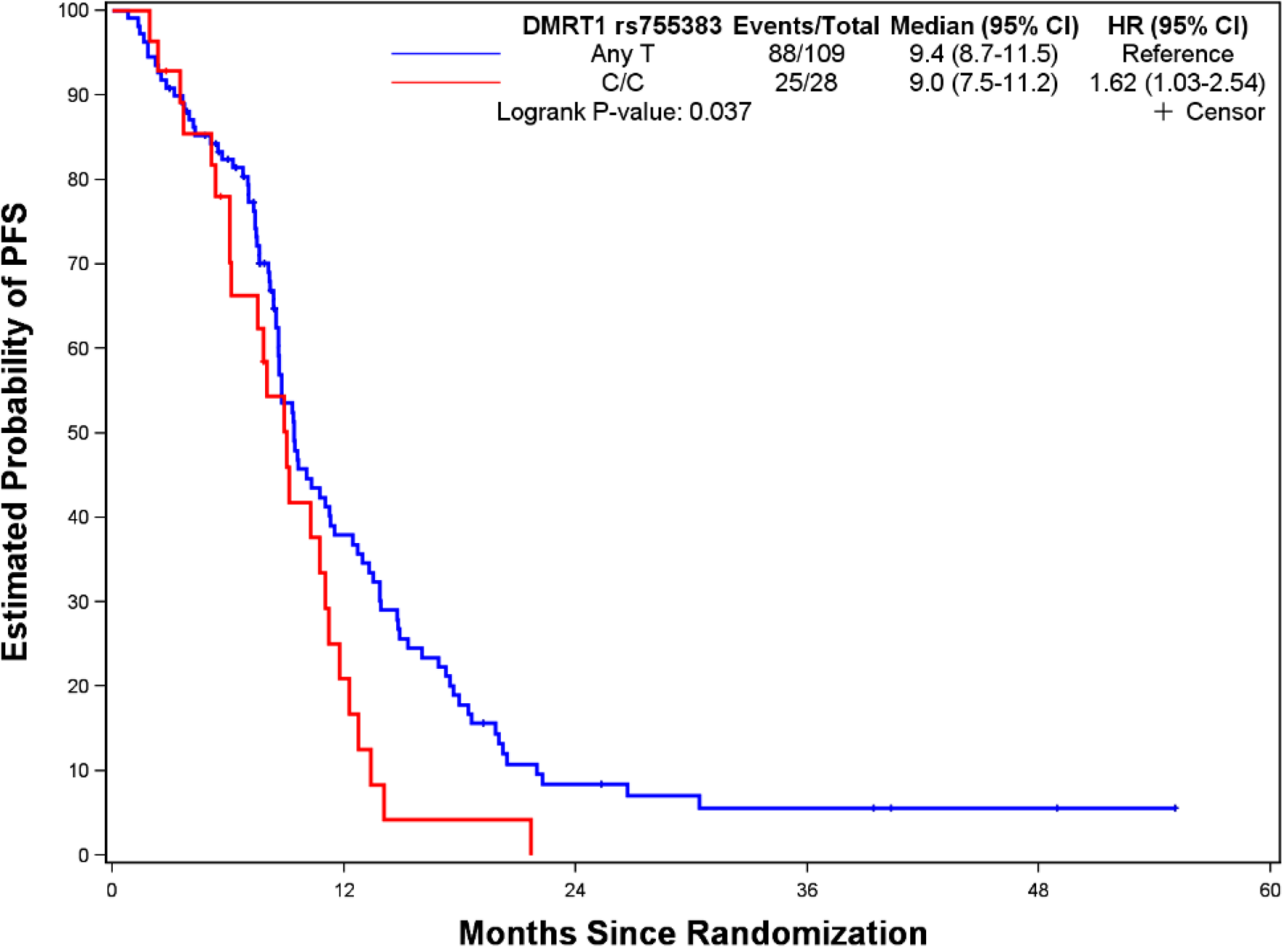
Kaplan–Meier curve and log-rank test for sex differentiation pathway SNP predictive of PFS in KRAS mutant mCRC patients. Patients with DMRT1 rs755383 Any T carriers have longer PFS (9.4 vs. 9.0 mo; *p* = 0.037) compared to C/C carriers.

*DMRT1* rs755383 was one of top predictors for PFS in KRAS mutant patients in RSF analysis. Patients with C/C variant had shorter PFS than those with Any T allele (*p* = 0.037) (Fig. 8). Whereas the previous findings based on FIRE3 study showed this SNP to be significant for PFS in KRAS wildtype patients^13^.

#### Tumor budding pathway SNPs

**Figure 9:**
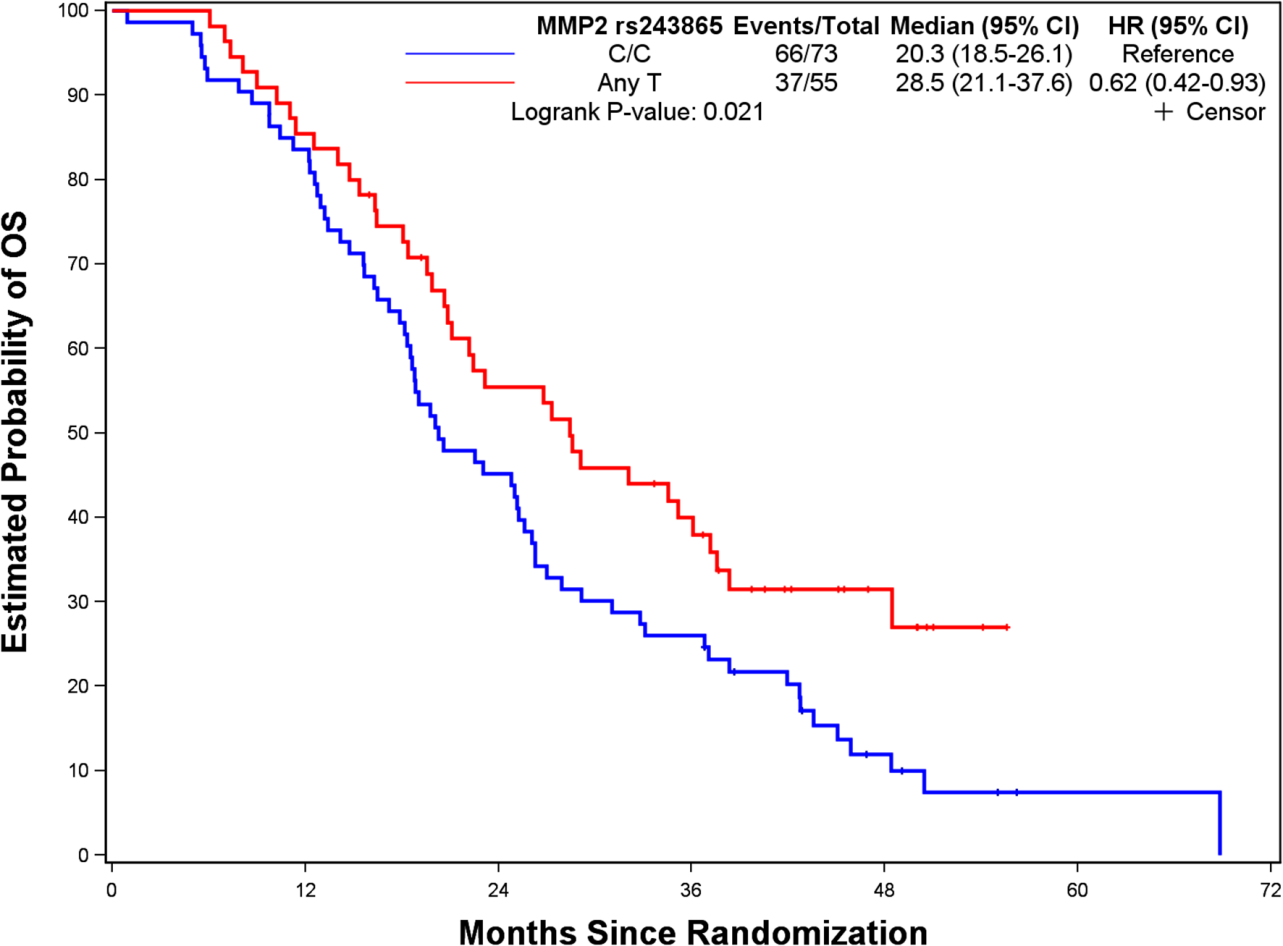
Kaplan–Meier curve and log-rank test for tumor budding pathway SNP predictive of OS in KRAS wild-type mCRC patients. Patients with MMP2 rs243865 Any T carriers have longer OS (28.5 vs. 20.3 mo; *p* = 0.021) compared to C/C carriers.

In our current study, a SNP in the tumor budding pathway, *MMP2* rs243865, was a strong predictor of OS in KRAS mutant patients. KM plot showed that patients with any T had a longer OS than those with C/C variant (*p* = 0.021, Fig. 9). This SNP was not identified as a predictive marker in our previous analysis in the bevacizumab-based chemotherapy treated patients from TRIBE arm A and PROVETTA trial^13^.

## Discussion

Our previous studies were mostly focused on candidate polymorphisms selected from a single pathway, and tested their association with clinical outcomes in a single treatment arm from a randomized clinical trial. These analyses were usually criticized with low statistical power. In this study, we combined the same treatment arms from two independent clinical trials, and performed random survival forest analysis for candidate SNPs from different pathways. Random forest has been applied broadly as a traditional machine learning method for classification and regression, and RSF is a new extension of RF to survival outcome data. It has been applied in several real-world studies such as GWAS study^14^ and systolic heart research^15^. The conventional regression-based methods to analyze survival data usually rely on restrictive assumptions such as proportional hazards in CPH regression models. In addition, the identifying interactions between variables is a common problem when building the regression model. In contrast, RSF method can handle these difficulties automatically using ensembled survival trees. RSF randomly draws bootstrap sample from data to grow survival tree, and at each node, a subset of randomly selected variables is chosen as candidate variables to split the truck. As such this method does not need to select candidate variables in advance like conventional methods. In our study, the general prediction accuracy of RSF models is higher than the conventional CPH models. In KRAS wildtype patients, the OOB C-indexes for PFS and OS are 0.50 and 0.44, respectively from RSF, compared to 0.32 and 0.39 from CPH models; in KRAS mutant patients, the C-indexes are 0.55 and 0.45 for PFS and OS, respectively from RSF, compared to 0.55 and 0.33 from CPH models.

High-throughput genomic technologies, such as single nucleotide polymorphism arrays have revolutionized CRC research by making it possible to identify biomarkers across the genome. However, detecting meaningful signals and making appropriate inferences from these massive datasets continues to pose challenges because of the high dimensionality and complex gene–gene interactions. We believe these complex gene–gene interactions underlie the poorly understood differences between KRAS wildtype and KRAS mutant mCRC patients. In this study, we used the high-dimensional power of RSF to identify pathways which could serve as predictive biomarkers in mCRC depending on KRAS status.

This is the first study to report that SNPs in the Wnt/β-catenin and TAM pathways are strongly predictive of OS and PFS in mCRC patients treated with FOLFIRI/bevacizumab independent of KRAS status. Wnt signaling results in β-catenin translocating to the nucleus and recruiting cyclic AMP response-element binding protein (CBP) to generate an active transcription complex^16^. Once activated, it regulates target gene expression by binding to the T-cell factor (TCF)/lymphoid enhancer factor family of transcription factors^17^. Glycogen synthase kinase 3-β (GSK-3β) and a multi-protein complex consisting of AXIN, APC and Diversin regulate phosphorylation of β-catenin, which targets it for ubiquitination and degradation^18^. The balance between β-catenin stabilization and degradation maintains cellular homeostasis.

Aberrant regulation of the Wnt signaling pathway is an established mechanism of colorectal carcinogenesis, however, its association with KRAS status is controversial. In this study, CBP and β-catenin were predictive of OS and PFS independent of KRAS status. Our results are in contrast with previous reports showing that oncogenic KRAS signaling stimulates Wnt pathway, which in turn promotes intestinal tumor growth and invasion^19,20^. Although KRAS hyperactivates Wnt signaling via TAK1 kinase^21^, there is considerable heterogeneity in the accumulation of nuclear β-catenin, suggesting the role of alternate pathways in influencing β-catenin distribution and Wnt pathway activation in CRC^19^.

Our study shows the close proximity of TAM and Wnt pathway SNPs on the minimal depth plots, which could highlight a possible interaction between the two pathways. Macrophages have two main phenotypes, M1 (tumor suppressive) and M2 (tumor-promoting and angiogenic)^12^. TAMs are derived from circulating monocytes, which upon recruitment to the tumor microenvironment, adopt the M2 phenotype and orchestrate conditions influencing tumor development^22^. The monocyte chemotactic protein-1, or CCL2, regulates polarization of M1 and M2 phenotypes and recruits TAMs to the tumor microenvironment^23^. CCL2 has been identified as a downstream target of β-catenin^23^. Furthermore, in vitro studies have shown that colon cancer cells stimulate macrophages to release IL-1β, which in turn enhances Wnt signaling in colon cancer cells, generating a self-amplifying loop promoting tumor growth^24^.

Macrophage activation is also regulated by TBK1, which is a noncanonical IkB and Tank-binding kinase-1, which activates IFN regulatory factor 3 (IRF3) and NF-kB-dependent genes^25^. Our current study showed that TBK1rs7486100 was strongly predictive of OS and PFS independent of KRAS status. TBK1 directly phosphorylates Akt signaling, and is an important downstream effector of KRAS^26,27^. TBK1 is shown to be essential in some human cancer cell lines with KRAS mutations^28^. Here, Barbie et al. show that suppression of TBK1 inhibited tumor formation in KRAS mutant cells, whereas suppression of TBK1 did not affect the tumorigenicity of KRAS wildtype colon cancer cells. Some studies have suggested the role of GSK-3β in activating TBK1^29^, which would present another interaction between the Wnt and TAM pathways. TBK-1 has also been shown to contribute to resistance to EGFR inhibitors via NF-κB signaling. Once TBK-1 activates NF-κB, this helps integrin avβ3 to confer cell resistance to EFGR inhibitors, the exact mechanism of which is currently unknown^30^. Hence, the strong predictive power of Wnt and TAM pathway SNPs in mCRC may lie in their complex interactions modulating the tumor microenvironment and angiogenesis, resulting in similar results observed in KRAS wildtype and KRAS mutant subgroups treated with bevacizumab-based chemotherapy.

Our RSF analysis identified DMRT1rs755383 SNP in the sex-differentiation pathway to strongly predict PFS in KRAS mutant patients. Polymorphisms in genes regulating sex differentiation have been shown to be predictive of PFS and OS in KRAS mutants^31^. KRAS mutant colorectal cancers show enhanced cancer stem cell pathways which accelerate tumorigenesis^32^. This is the first study to report a relationship between the sex differentiation gene, DMRT1 and KRAS mutant in mCRC.

DMRT1 (doublesex and mab-3-related transcription factor 1) is a sex determination gene located on chromosome 9^27^, which serves as a tumor suppressor^33^. It encodes a transcription factor that plays a key role in male sex determination and differentiation by controlling testis development and male germ cell pluripotency^34^. In humans, several deletions in chromosome 9 have been associated with sex reversal and gonadal dysgenesis in XY individuals^35^. DMRT1rs755383 is associated with the development of testicular germ cell tumors^36^. The DMRT1 pathway is intertwined with Wnt signaling, where Wnt activated SOX9 which activates DMRT1^37^. DMRT1 further inhibits SOX2, which is associated with a cancer stem cell state in colorectal cancer^38^. There are many reports that Wnt/β-catenin pathways play important roles in the maintenance of cancer stem cells^39^, which help confer resistance to EGFR inhibitors^40^. Cancer cells with stem-cell properties develop resistance against tyrosine kinase inhibitors by expressing drug transporting proteins such as the ATP-binding cassette family (ABC) and facilitating epithelial-to-mesenchymal (EMT) transition. Hence, a stem cell state observed in KRAS mutant CRCs may be related to its relationship with DMRT1, which warrants further investigation as a target for drug development.

Our study had a few limitations. Firstly, differences in the locations and types of KRAS mutations between the two trials could have affected our results. Secondly, patients in FIRE-3 were only selected for KRAS exon 2 wildtype status based on clinical evidence of response to cetuximab, and therefore, this cohort did not include alternate KRAS codons with wildtype status. Although KRAS exon 3 and 4 mutations are associated with resistance to EGFR inhibitors, they interact with different pathways. For instance, exon 4 mutations are known to have MEK pathway dependence^41^, which was not studied in our population. Hence, it is possible our study did not capture the heterogeneity in clinical outcomes due to the diversity of KRAS mutations. In addition, to increase the statistical power, we combined two independent trials, while the genetic difference may exist between two cohorts. Lastly, both trials included Caucasian patients, therefore, our results cannot be applied to an ethnically diverse population. The results of this study need to be validated in different patient populations.

It is important to note that the relationship between these pathways and KRAS is non-linear and involves complex interactions between multiple agents, including the tumor microenvironment, epigenetic regulation, multiple polymorphisms and unique cell physiology. This study offers a unique approach to exploring relationships and interactions between multiple pathways, but the complex nature of the KRAS pathway cannot be understood by focusing on one pathway alone.

In summary, RSF is a useful method of identifying pathway interactions in high-dimensional settings to derive outcome data. In this study, we applied RSF to understand the genomic relationships between KRAS wildtype and mutant mCRC patients. We discovered that Wnt and TAM pathway SNPs might interact with each other to predict OS and PFS in mCRC treated with FOLFIRI/bevacizumab independent of KRAS status, whereas DMRT1 SNP may be an important predictive marker in KRAS mutant patients. Our results suggest new pathways predictive of survival in the KRAS subgroups, and further understanding of these relationships may be useful for developing improved targeted treatments.
